# Phylogenetic analysis of eight sudanese camel contagious ecthyma viruses based on B2L gene sequence

**DOI:** 10.1186/s12985-015-0348-7

**Published:** 2015-08-12

**Authors:** Abdelmalik I. Khalafalla, Ibrahim M. El-Sabagh, Khalid A. Al-Busada, Abdullah I. Al-Mubarak, Yahia H. Ali

**Affiliations:** Camel Research Center, King Faisal University, Al-Ahsa, 31982 Saudi Arabia; Department of Microbiology, Faculty of Veterinary Medicine, University of Khartoum, P.O. Box 32, Shambat, Sudan; Central Biotechnology Laboratory, Faculty of Veterinary Medicine and Animal Resources, King Faisal University, Al-Ahsa, 31982 Saudi Arabia; Department of Virology, Cairo University, Giza, 12211 Egypt; Department of Biochemistry, Physiology and Pharmacology, College of Veterinary Medicine and Animal Resources, King Faisal University, Al-Ahsa, 31982 Saudi Arabia; Department of Microbiology and Parasitology, College of Veterinary Medicine and Animal Resources, King Faisal University, Al-Ahsa, 31982 Saudi Arabia; Department of Virology, Central Veterinary Research Laboratory, P. O. Box 8067, Al Amarat, Khartoum, Sudan

**Keywords:** Camel contagious ecthyma, B2L gene, Phylogenetic analysis

## Abstract

**Background:**

Camel contagious ecthyma (CCE) is an important viral disease of *camelids* caused by a poxvirus of the genus *parapoxvirus* (PPV) of the family *Poxviridae*. The disease has been reported in west and east of the Sudan causing economical losses. However, the PPVs that cause the disease in camels of the Sudan have not yet subjected to genetic characterization. At present, the PPV that cause CCE cannot be properly classified because only few isolates that have been genetically analyzed.

**Methods and results:**

PCR was used to amplify the B2L gene of the PPV directly from clinical specimens collected from dromedary camels affected with contagious ecthyma in the Sudan between 1993 and 2013. PCR products were sequenced and subjected to genetic analysis. The results provided evidence for close relationships and genetic variation of the camel PPV (CPPV) represented by the circulation of both Pseudocowpox virus (PCPV) and Orf virus (ORFV) strains among dromedary camels in the Sudan. Based on the B2L gene sequence the available CPPV isolates can be divided into two genetic clades or lineages; the Asian lineage represented by isolates from Saudi Arabia, Bahrain and India and the African lineage comprising isolates from the Sudan.

**Conclusion:**

The camel parapoxvirus is genetically diverse involving predominantly viruses close to PCPV in addition to ORFVs, and can be divided into two genetically distant lineages. Based on sequences of the B2L gene it is not possible to suggest that the viruses that cause CCE form a monophylogenetic group or species within the PPV phylogeny.

## Background

Camel contagious ecthyma (CCE), also known as Auzdik, Orf in camels and Pustular dermatitis is a contagious skin disease of *camelids* caused by a pox virus of the genus *parapoxvirus* (PPV), subfamily *Chordopoxvirinae* of the family *Poxviridae*. The disease has a worldwide distribution and has been reported in Mongolia [1], Kenya [2], Kazakhstan and Turkmenistan [3], Somalia [4], Sudan [5–7], Libya [8], Saudi Arabia [9, 10], Bahrain [11] and India [12]. The disease is endemic in affected areas with variations in intensity of infection, morbidity and mortality rates and tends to occur annually in the rainy season, as the situation in Sudan and Saudi Arabia, affecting mostly young animals. The age group at risk are those less than one year of age [6] including cases reported in month-old camel calves in Saudi Arabia [10]. The marked seasonality associated with the rainy season may be due to the optimum condition for the survival and perpetuation of the virus and skin abrasions caused by browsing thorny trees [7]. In most cases, the disease caused no mortality, but when camel calves are severely affected the pox-lesions interferes with the calves’ ability to suckle or graze and extends to eyelids leading to blindness, particularly at the Savanah belt in the Sudan, leading to mortality rates that can reach 9 % [7]. Clinically, the pox-lesions first appeared on the lips of affected animals as small papules that progressively developed into scabs on the lips, muzzle, nares and eyelids culminating into fissured crusts on the lips. Swelling of the head and sometimes the neck have been observed in the field. The lesion is proliferative and highly vascularized and may extend into gum, palate and tongue. The major factors associated with increased likelihood of CCE occurrence are season of the year, camel age, camel movements and location and their association with thorny trees [7].

Camel contagious ecthyma is a sparsely studied disease and the causative virus have only recently genetically characterized. According to Abubakr et al [11] sequence homologies and phylogenetic analysis of the major envelope gene (B2L) of 2 CCE skin scabs (two from Bahrain and one from Saudi Arabia) showed that these viruses are closely related to the pseudocowpox virus (PCPV) species of the PPV genus of the family *poxviridae*. In addition, Nagarajan et al [12] showed that CCE in Indian dromedary camels is also caused by a PCPV.

Currently, there are four established species within the PPV genus of the family *Poxviridae*; Orf virus (ORFV), the type species of the genus, which causes disease mainly in sheep and goats, PCPV and bovine papular stomatitis virus (BPSV) both infect cattle, in addition to Parapoxvirus of red deer in New Zealand (PVNZ), which has only been isolated from red deer in New Zealand. BPSV and PCPV affect mainly cattle, but differ from ORFVs in the site of the pox lesion, as BPSV is restricted to the muzzle and PCPV the teat. Tentative species include the camel PPV (CPPV), reindeer parapox virus, musk ox and seal parapoxvirus [13–15]. At present, the CPPV that cause CCE cannot be properly classified because only few isolates that have been genetically analyzed.

Camel contagious ecthyma is believed to be endemic in the Sudan since long time, but was first diagnosed in the disease outbreaks west of the country in 1986 [5]. Later on, the disease was reported in eastern Sudan [6, 7]. However, the PPVs that cause the disease in dromedary camels of the Sudan have not yet subjected to genetic characterization.

In the present study, we investigated lesion-derived DNAs from eight skin scabs collected from dromedary camels in the Sudan presented with CCE lesions and showed for the first time, that the CPPV does not form a monophyletic clade within the PPV phylogeny and the disease is caused by viruses from more than one PPV species.

## Results

**Fig. 1:**
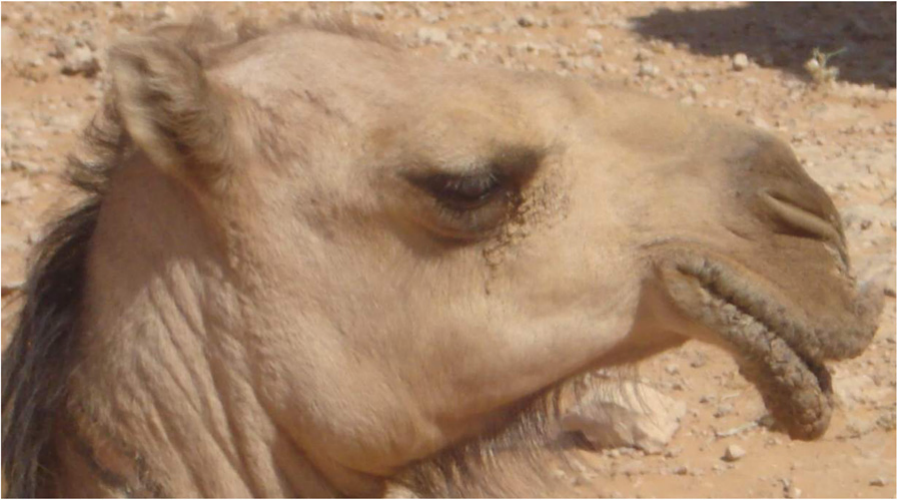
Contagious ecthyma lesions on the lips of a 14 month-old dromedary camel, Sudan

In this investigation, we screened archive scab sample collected between 1993 and 2013 in addition to the new scab collection from Sudan in August 2013. The 2013 outbreak of the disease involved 17 camel herds around Showak area of eastern Sudan, causing morbidity and mortality rates of 20 and 1.5 %, respectively. Pustular dermatitis lesion characteristic for CCE were observed (Fig. 1). Out of 10 scab materials, eight gave positive specific amplification in real-time PCR and gel-based PCR (data not shown). Table 1 shows the origin of positive samples used in this study. To characterize the DNA fragments generated by PCR, the PCR products were sequenced and subjected to genetic analysis.

**Table 1:** Details of scab specimens collected from dromedary camels in Sudan

No	Sample	Date of collection	Area	Age of affected camel (month)
1	SD-V4	Aug. 1993	Showak, east Sudan	12
2	SD-V8	Aug. 1993	Showak, east Sudan	11
3	SD-V13	Sept. 1994	Obeid, west Sudan	13
4	SD-V34	Jul. 2000	Showak, east Sudan	10
5	SD-V20	Jul. 2005	Showak, east Sudan	10
6	SD-K1	Aug. 2013	Showak, east Sudan	12
7	SD-K2	Aug. 2013	Showak, east Sudan	11
8	SD-K3	Aug. 2013	Showak, east Sudan	11

The B2L gene fragment from the eight DNAs has a high G + C content of 62.6 to 63.9 % (Table 2). The seven isolates (SD-V4, V8, V13, V20, K1, K2 and K3) shared 96–98 % homology with viruses in the species PCPV while one isolate (V34-SD) shared 96–99 % homology with viruses in the ORFV species of the genus PPV (Table 2). Seven CPPV Sudanese isolates shared 96–98 % nucleotide sequence identity with virus strains in PCPV species, while one isolate (V34-SD) shared only 94–95 % identity with PCPV, but 96–99 % identity with ORFV.

**Table 2:** Nucleotide percent identity and G + C content based on B2L gene after BLAST search involving eight CPPVs analyzed in the present study

		Percent Identity with PCPV	Percent Identity with ORFV	G + C Content
1	SD-V4	96–79	92–94	62.6
2	SD-V8	97–98	93–94	63.2
3	SD-V13	96–98	92–94	63.0
4	SD-V20	97–98	92–94	63.0
5	SD-V34	94–95	96–99	63.9
6	SD-K1	97–98	92–94	63.0
7	SD-K2	96–98	92–94	62.7
8	SD-K3	96–98	92–94	63.0

Results of multiple sequence alignment and per cent identity matrix based on 160 amino acids sequences (Table 3) revealed that the Sudanese CPPVs from 1993 to 2013 examined in the present study, shared variable amino acid sequence identity ranged from 95.04 to 100 % (intra-group similarity). Five isolates (V4-SD, V20-SD, K1-SD, K1-SD and K3-SD) possessed 100 % identity, isolate V13-SD has a less homology of 95.04 to 99.69 % with the rest Sudanese isolates. On the other hand, isolate V34-SD, which has the closest identity to ORFV species, shared a relatively lower per cent identity of 95.04–96.21 with the rest seven isolates (Table 3). As shown in Table 3, the previously published five CPPV isolates Judhpur-IN and Cam/09 from India, BH-1 and BH-3 from Bahrain and SA-98 from Saudi Arabia shared intra group similarity of 98.94 to 100 % homology and a 95.04 to 99.69 % homology with the eight Sudanese isolates analysed in the present study. On the other hand, isolate CE41, which was classified as BPSV shared only 81.21 to 82.55 % homology with all the CPPVs.

**Table 3:** Percent Identity Matrix based on 160 amino acids created by Clustal 2.1 for all published CPPV B2L gene sequences

		1	2	3	4	5	6	7	8	9	10	11	12	13	14
1	Jodhpur-IN		98.94	99.38	99.38	99.38	81.88	95.04	99.40	99.69	99.74	99.69	100.0	99.69	99.69
2	Cam/09	98.94		100.0	100.0	100.0	81.21	95.04	99.40	99.69	99.21	99.69	99.39	99.69	99.69
3	1-BH	99.38	100.0		100.0	100.0	81.21	95.45	98.75	99.38	99.38	99.38	99.38	99.38	99.38
4	3-BH	99.38	100.0	100.0		100.0	81.21	95.45	98.75	99.38	99.38	99.38	99.38	99.38	99.38
5	98-SA	99.38	100.0	100.0	100.0		81.21	95.45	98.75	99.38	99.38	99.38	99.38	99.38	99.38
6	CE41	81.88	81.21	81.21	81.21	81.21		84.85	82.55	81.88	81.88	81.88	81.88	81.88	81.88
7	V34-SD	95.04	95.04	95.45	95.45	95.45	84.85		95.04	95.42	95.42	95.42	96.21	95.42	95.42
8	V13-SD	99.40	99.40	98.75	98.75	98.75	82.55	95.04		99.69	99.70	99.69	99.39	99.69	99.69
9	V8-SD	99.69	99.69	99.38	99.38	99.38	81.88	95.42	99.69		100.0	100.0	100.0	100.0	100.
10	K2-SD	99.74	99.21	99.38	99.38	99.38	81.88	95.42	99.70	100.0		100.0	100.0	100.0	100.
11	K1-SD	99.69	99.69	99.38	99.38	99.38	81.88	95.42	99.69	100.0	100.0		100.0	100.0	100.
12	V4-SD	100.00	99.39	99.38	99.38	99.38	81.88	96.21	99.39	100.0	100.0	100.0		100.0	100.
13	V20-SD	99.69	99.69	99.38	99.38	99.38	81.88	95.42	99.69	100.0	100.0	100.0	100.0		100.
14	K3-SD	99.69	99.69	99.38	99.38	99.38	81.88	95.42	99.69	100.0	100.0	100.0	100.0	100.0	

**Fig. 2:**
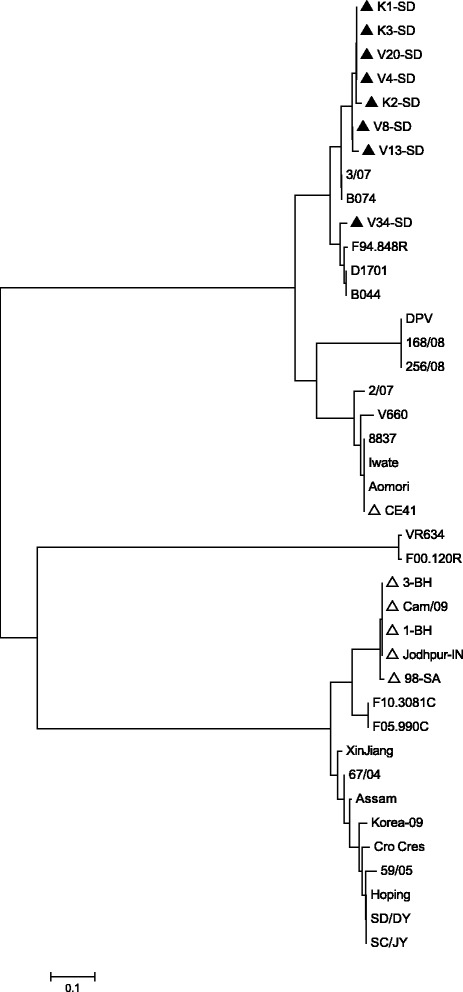
Molecular Phylogenetic analysis of 40 PPV amino acid sequences based on B2L gene. The evolutionary history was inferred using the Neighbor-Joining method [16]. The optimal tree with the sum of branch length = 3.16641726 is shown. The tree is drawn to scale, with branch lengths in the same units as those of the evolutionary distances used to infer the phylogenetic tree. The evolutionary distances were computed using the Poisson correction method [17] and are in the units of the number of amino acid substitutions per site. The analysis involved 40 amino acid sequences. All positions containing gaps and missing data were eliminated. There were 85 positions in the final dataset. Evolutionary analyses were conducted in MEGA6 [18]. Black triangle dots represent the eight Sudanese CPPV isolates analyzed in the present study and empty triangles CPPV published sequences

A phylogenetic tree was created for the amino acids sequences of the B2L gene of 40 PPV strains, including the eight strains described in the present study using the Neighbor-Joining method. The result revealed that the Sudanese CPPVs clustered with other PPVs published earlier and that the analyzed PPVs displayed distinct clusters representing BPSV and PVNZ species (Fig. 2). Members of the other two PPV species, namely ORFV and PCPV are distributed between the two main branches shown in the phylogenetic tree. Seven of the eight Sudanese CPPV isolates sequenced in the present study clustered together closer to PCPV isolates from Finland and Germany. One Sudanese isolate (V34-SD) clustered in a different sub-branch that contains ORFV isolates from Finland and Germany and a PCPV isolate from Finland. On the other hand, the five previously published sequences representing CPPV isolates from India, Saudi Arabia and Bahrain clustered at the other branch of the phylogenetic tree together with PCPV isolates from Finland and ORFV isolates from Asian countries (India, Korea, China and Taiwan). Isolate CE41 isolated from a dromedary camel in the Sudan clustered with the BPSV species of PPV.

The obtained nucleotide sequences were submitted to GenBank and assigned the accession numbers KR231664, KR231665, KR231666, KR231667, KR231668, KR231669, KR231670 and KR231671 for isolates V4-SD, V8-Sd, V13-Sd, V20-SD, V34-SD, K1-SD, K2-SD and K3-SD, respectively.

## Discussion

A preferred target gene to generate PCR amplified DNA fragments for sequence analysis and comparison of PPV DNA is the open reading frame (ORF) 011 (B2L gene), the PPV homologue of the vaccinia virus Copenhagen (VACV) gene F13L, which encodes the major envelope antigen p37K [19].

In the present study, a genetic analysis of eight Sudanese isolates of the CPPV was performed. BLAST results unveiled that seven isolates are genetically nearly related to PCPV and one is closer to ORFV. The B2L gene fragment from the eight DNAs has a high G + C content of 62.6 to 63.9 % in agreement with that of the whole PPV genome [20].

The findings of the present study provide evidence for a close relationship of seven Sudanese strains despite the different geographic location and year of collection. Interestingly, five isolates collected at the same area of eastern Sudan, but during a time span between 1993 and 2013 have 100 % amino acid homology pointing to a circulation of genetically stable virus and a stable genetic makeup and a wide distribution in the country.

The dominant CPPV circulating in the Sudan is found to be genetically close to PCPV species of the PPV and a genetic variability in this country, of at least two PPV species: PCPV (*n* = 7) and ORFV (*n* = 1). Three isolates, which were collected over 20 years, possessed 100 % genetic homology.

Search of the literature showed that five isolates have been antecedently classified as PCPV [11, 12]. The results of this study ascent the total number of CPPV that belong to PCPV species to 12. However, the study also identified one isolate that genetically closer to ORFV than PCPV. Besides, we also corroborated previous classification of isolate CE 41 as BPSV [21]. Taken together, the present study shows heterogeneity among the CPPVs and suggest that clearly, these viruses do not form a monophyletic species within PPV phylogeny, at least based on the B2L gene analysis. A search of the literature revealed the presence of only one CPPV strain (Cam/09) that has been classified as ORFV (accession # GU460370.1) as it has 99 % nucleotide homology with an Indian ORFV from sheep [22]. Here we confirm previous research work and present grounds that CCE in camels can be caused by ORFV. This is what has been anticipated before any genetic analysis was made, as lesions produced by the causative virus is characterized by a progressive nodule-vesicle-pustule-scab formation found mainly on the lips that resembles lesions induced by ORFV in sheep and goats, but not PCPV or BPSV [9]. The findings also might points to a transmission from sheep or goats, as it is common for camels, sheep, and goats to share the same pasture in the Sudan. Contrary to such speculation, Abu Elzein et al [9], in Saudi Arabia failed to experimentally infect sheep and goats with an isolate of CPPV. Unfortunately, the PPV circulating in sheep and goats in the Sudan has not yet genetically analyzed. Therefore, molecular analysis of ORFV circulating in small ruminants in the same area and experimental infection of sheep and goats with a CPPV genetically closer to ORFV is expected to rule out such possibility.

Previous reports from Saudi Arabia [11] and India [12] implicated PCPV as the cause of the disease in camels. Of note, Abubakr et al [11] on the bases of their first connection of the CPPV to PCPV postulated a relatively recent introduction of the virus to camels, possibly from humans, that have been exposed to PCPV-infected ruminants. Yet, no PCPV infection in human or animals has been reported in the Sudan or Arabian Peninsula, but only one case of ORFV infection of a man in United Arab Emirates [23]. Of interest is to determine the reservoir host that maintain the virus in nature during the inter-epizootic period. A serological survey involving domestic and wild species of animals in the affected area of eastern Sudan may identify such reservoir host and contribute to its control.

On the other hand, a nucleotide sequence of the B2L gene from a cell culture- adapted CPPV (CE41) was found to be genetically closely related to BPSV [21]. This genetic placement might be caused by genomic rearrangements known to occur when PPVs are passaged in cell culture [24–26]. Nevertheless, taking into consideration the data presented in this report, it can be postulated that contagious ecthyma in dromedary camels is caused by viruses from different species of PPV. This situation is not uncommon as some PPVs obtained from wild Japanese serows are genetically closer to ORFV, and some are closer to BPSV [27]. However, a possibility still exists that a separate virus species of PPV genetically related to both PCPV and ORFV based on the sequence of the B2L gene, but differ in other genomic domains causes this disease. To this end whole genome sequencing and comparison with known PPV species is a requisite and anticipated to bring out a better approximation to reality.

At present 11 out of the 14 published CPPV sequences are grouped along with PCPV, two are ORFV related and one belonged to the BPSV species of PPV indicating genetic variability of the CPPV. Further investigations should examine other genes of the virus and perform complete genome sequencing in order to better describe and classify the CPPV.

According to previous phylogenetic analysis based on the B2L gene of PPV, PCPV and ORFV strains are usually characterized by a certain level of variability, while PVNZ and BPSV strains may possess 100 % identity [21]. In line with that, the phylogenetic tree generated in this study for 40 PPV sequences showed distinct clustering of BPSV and PVNZ strains, with ORFV and PCPV strains bundling in more than one subgroup mostly with a link to the geographic location of respective disease outbreaks. Similarly Oem et al [28] showed that some PPVs of a particular classification clustered with different species in the phylogenetic tree. Therefore, the present study supports the contention by Oem et al [28] that phylogenetic analysis using partial B2L gene sequences may not be suited owing to the recent increase in the publication of more sequences of PPV from different countries and hosts. Besides, it should also be kept in mind that infections with multiple PPV strains of a single species of PPV can occur in one animal as recently described [29].

CCE is a sparsely studied disease of camels and presently does not have to be reported to the World Organization of Animal Health (OIE) despite several publications reporting wide distribution of the disease. The disease is economically important and a recent publication from Iran [30] described severe cases of CCE that caused a mortality rate of 6 %, therefore, this important camel disease has to be looked at seriously.

## Conclusion

We genetically analyzed based on sequences of the B2L gene eight strains of CPPV collected from two areas of the Sudan between 1993 and 2013. The prevailing virus is genetically close to PCPV species of the PPV and a genetic variability in this country, of at least two PPV species exists: PCPV (*n* = 7) and ORFV (*n* = 1). Based on sequences of the B2L gene it is not possible to suggest that the viruses that cause CCE form a monophylogenetic group or species within the PPV phylogeny.

## Methods

### Origin of specimens

This study was approved by the Institutional Review Board of the Veterinary Research Institute, Sudan. This study was approved by the Institutional Review Board of the Veterinary Research Institute, Sudan Skin scab specimens (*n* = 6) were collected from dromedary camels (*camelus dromedarius*) showing symptoms of contagious ecthyma in east and west Sudan during field epidemiological investigations between 1993 and 2005. Diagnosis of the disease was made on the bases of clinical evidence and electron microscopy and later by PCR [31]. Details on disease symptoms and epidemiology were published [6]. Additional four specimens were collected in 2013. Data on the clinical picture, morbidity and mortality rates resulted in the 2013 disease outbreaks in Showak area of eastern Sudan were collected. Table 1 shows some data on positive specimens analyzed in the present study.

### Tissue homogenization and DNA extraction

A 20 % suspension (weight/volume) was made of the scab material in tris-EDTA (TE) buffer (pH 7.4), freeze-thawed at −30 °C, mechanically homogenized using a mechanical Homogenizer (TissueRuptor, Qiagen, Hilden, Germany) and centrifuged at 1500 × g for 10 min at 4 °C. Total viral DNA was extracted from supernatants using a QiaAmp DNA Mini Kit (Qiagen, Hilden, Germany) according to manufacturer’s instructions.

### PCR

To check its PPV identity each extracted DNA was first tested using the quantitative real-time PCR assay described by Nitsche et al [32]. The amplification was carried out on Mx3000P qPCR system (Agilent Technologies, USA) using Brilliant II QPCR Master Mix (Agilent Technologies, USA). Second, with DNA of known PPV identity, a PCR protocol was used to obtain DNA fragment for sequencing. This PCR assay targets the commonly used B2L gene (open reading frame 011) which is homologue of vaccinia virus Copenhagen (VACV) gene F13L encoding the major envelop antigen p37K [19]. PCR amplification was done using forward primer (5′-TTAATTTATTGGCTTGCAGAACTCCGAGCGC-3′) and reverse primer (5′-ATGTGGCCGTTCTCCTCCATC-3′) [33] that amplify 1200 bp DNA sequence. PCR amplification of the envelope gene was performed using the following thermal profiles initial denaturation at 94 °C for 10 min, followed by 35 cycles of denaturation at 94 °C for 1 min, annealing at 55 °C for 30 s, extension at 72 °C for 1.5 min, and final extension at 72 °C for 10 and DNA was amplified with a thermal cycler TP3000 (Biometra, Germany). The PCR products were then checked in agarose gel, purified using QIAquick PCR Purification Kit (Qiagen, Hilden, Germany) and then sent for sequencing. Sequencing was completed using the BigDye® Terminator v3.1 cycle sequencing kit chemistry and each primer pairs. Nucleotide positions were confirmed based on two independent sequencing reactions in both directions.

### BLAST and phylogenetic analysis

The terminal unreliable nucleotides of the obtained B2L gene sequences were first trimmed-off automatically and both forward and reverse sequences aligned in the Geneious® 8.1.4 package. The biologically correct sequences of the eight CPPV isolates were subjected to basic local alignment search was compared with nucleotide sequence in the GenBank database using the online BLASTN program on the NCBI website [34].

Phylogenetic tree based on amino acid sequences was constructed for 40 PPV strains (Table 4), including sequences from the four PPV species; ORFV (*n* = 9), PCPV (*n* = 10), BPSV (*n* = 6) and PVNZ (*n* = 3) and eight strains from this study, using the Neighbor-Joining method in MEGA6 [18]. The amino acid substitution model used was the Johen-Tylor-Thornton model [45]. The significance of all deduced phylogenetic trees was verified by bootstrap analysis of 1000 replicates.

**Table 4:** Information on Parapoxviruses used for the phylogenetic analysis of the B2L gene

No	Species	Virus identification	Host	Origin	GenBank accession	Reference
1	ORFV	D1701	Reindeer	Finland	AY453654.1	Tikkanen et al (2004) [35]
2	ORFV	Korea 09	Goat	Korea	GQ328006.1	Oem et al (2013) [28]
3	ORFV	59/05	Sheep	India	DQ263304.1	Hosamani et al (2006) [36]
4	ORFV	Hoping	Goat	Taiwan	EU935106.1	Chan et al (2009) [37]
5	ORFV	B044	Goat	Germany	KF478798.1	Friederichs et al (2014) [38]
6	ORFV	Cam/09	Dromedary	India	GU460370.1	Venkatesan (unpublished)
7	ORFV	Assam	Goat	India	JQ040300.1	Bora et al. (2012) [39]
8	ORFV	Xinjiang	Sheep	China	JN565694	Li et al. (2013) [22]
9	ORFV	Jodhpur	Dromedary camel	India	GQ390365	Nagarajan et al (2010) [12]
10	ORFV	SD/DY	Sheep	China	JQ904794.1	Zhang et al (2014) [40]
11	ORFV	Cro_Cres	Sheep	Croatia	HQ215589.1	Lojkic et al (2010) [41]
12	ORFV	SC/JY	Goat	China	JQ904792.1	Zhang et al (2014) [40]
13	ORFV	67/04	Sheep	India	DQ263305.1	Hosamani et al (2006) [36]
14	PCPV	F94.848R	Reindeer	Finland	AY453661.1	Tikkanen et al (2004) [35]
15	PCPV	F05.990C	Reindeer	Finland	JF773694.1	Hautaniemi et al (2011) [42]
16	PCPV	F10.3081C	Reindeer	Finland	JF773695.1	Hautaniemi et al (2011) [42]
17	PCPV	3/07	Cattle	Germany	KF478804.1	Friederichs et al (2014) [38]
18	PCPV	B074	Man	Germany	KF478803.1	Friederichs et al (2014) [38]
19	PCPV	SA 98	Dromedary camel	Saudi Arabia	EF555793.1	Abubakr et al (2007) [11]
20	PCPV	BH 1	Dromedary camel	Bahrain	EF555792.1	Abubakr et al (2007) [11]
21	PCPV	BH 3	Dromedary camel	Bahrain	EF555791.1	Abubakr et al (2007) [11]
22	PCPV	F00.120R	Reindeer	Finland	GQ329669	Hautaniemi et al (2011) [42]
23	PCPV	VR634	Man	New Zealand	GQ329670.1	Gassmann et al. (1985) [43]
24	BPSV	V660	Cattle	Germany	KF478805.1	Friederichs et al (2014) [38]
25	BPSV	2/07	Cattle	Germany	KF478806.1	Friederichs et al (2014) [38]
26	BPSV	CE 41	Dromedary camel	Sudan	JN171861.1	Dal Pozzo et al. (2011) [21]
27	BPSV	Aomori	Cattle	Japan	AB044797.1	Inoshima et al. (2001) [27]
28	BPSV	8837	Cattle	Korea	JX968998.1	Oem et al. (2013) [28]
29	BPSV	Iwate	Cattle	Japan	AB538385	Oem et al (2013) [28]
30	PVNZ	DPV	Red Deer	New Zealand	AY453655.1	Tikkanen et al (2004) [35]
31	PVNZ	168/09	Red Deer	Italy	HQ239068.1	Scagliarini et al (2011) [44]
32	PVNZ	256/08	Red Deer	Italy	HQ239069.1	Scagliarini et al (2011) [44]
